# Correction: Effects of gender sensitive language in job listings: A study on real-life user interaction

**DOI:** 10.1371/journal.pone.0329656

**Published:** 2025-08-04

**Authors:** Dominik Hetjens, Stefan Hartmann

The Data Availability statement for this article is incorrect. The correct statement is: The full data and analysis scripts can be found at https://doi.org/10.17605/OSF.IO/Q986X.
