# Supplementary material for: Effects of gender sensitive language in job listings: A study on real-life user interaction
Source: PLoS One. 2024 Aug 14;19(8):e0308072. doi: 10.1371/journal.pone.0308072 (PMC11324110; doi:10.1371/journal.pone.0308072)
Supplement: Attachment — Submitted filename: Effects of gender sensitive language in job listings_review.pdf [file pone.0308072.s001.pdf]

Review letter

Effects of gender sensitive language in job listings: a study on real-life user interaction

The entire manuscript is exceptionally well-written, featuring a meticulously structured case study section, clear objectives, and a robust methodology. The study demonstrates a strong motivation, drawing from previous research, and the questions posed within this manuscript are pertinent. They have the potential to yield significant insights into the impact of gender-sensitive language, particularly in the context of job listings, which is an important and timely topic. This paper is poised to make substantial contributions across various fields, with the potential for significant impact. Moreover, it introduces a novel perspective by analyzing real language usage with a large sample size to explore gender-sensitive language, while also providing a methodology that can pave the way for further research inquiries. However, navigating through the manuscript, especially the introduction, can prove challenging. I believe that reorganizing and simplifying the content is necessary to facilitate comprehension for a wider audience.

I recommend that the author begins with a more general review of gender and gender sensitivity, along with their implications, before delving into the specific situation in German language. The current introduction may deter some readers, as it appears overly focused on the German context, whereas I believe it holds broader implications. While I appreciate the authors' thorough introduction, I suggest trimming it down and focusing on more specific points to avoid repetition. For instance, the method of specifying the feminine is mentioned multiple times through the addition of suffixes. Additionally, some of the reviewed studies contain excessive detail, such as including the professions of the authors, which may fatigue external readers. The introduction would benefit from being streamlined and more concise overall. It currently resembles a chapter from a book rather than an introduction to a paper.

The subsections on "Systemic Approach" and "Corpus-based Approach" incorporate various studies, theoretical frameworks, and underlying philosophies of each approach. While it's important to explain these to readers, they don't have a direct link to the main topic. However, the "Experimental Approach" includes specific studies relevant to job advertisements, which is the primary focus. It might be more effective for the author to begin with literature directly related to job advertisements and the experimental approach. Currently, readers have to wait until page 22 to encounter specific literature on the main topic. I recommend introducing this literature on job advertisements earlier, followed by a brief overview of how systemic and corpus-based approaches can aid in investigating this topic, emphasizing their relevance to gender studies. Each approach could be presented in a condensed and more focused manner. Additionally, since the study combines corpus-based and experimental approaches, less emphasis should be placed on systemic approaches.

In the case study section, it's challenging to discern which aspects of the current study and the analysis the authors consider part of the experimental approach versus the corpus-based approach. Since the main objective of the study is to capitalize on the strengths of both methodologies, it's crucial to clarify this explicitly. While it's apparent that classifying job listings based on genderism within the corpus analysis, and tracking the number of views by real users aligns more with the experimental approach, the distinction isn't sufficiently clear. This becomes especially important given the absence of additional data such as reaction times, acceptability tests, or eye-tracking, which are typically integral components of the experimental approach. Therefore, I suggest strengthening the separation between the two methodologies to provide greater clarity and understanding for readers.
